# PD1CD28 chimeric molecule enhances EGFRvⅢ specific CAR-T cells in xenograft experiments in mouse models

**DOI:** 10.1371/journal.pone.0310430

**Published:** 2024-10-01

**Authors:** Wanqiong Chen, Na Xian, Ningning Zhao, Qiong Zhang, Yunlu Xu

**Affiliations:** 1 School of Pharmacy, Quanzhou Medical College, Quanzhou, Fujian, China; 2 Institute of Immunotherapy, Fujian Medical University, Fuzhou, Fujian, China; 3 Tcelltech Biological Science and Technology Inc., Fuzhou, Fujian, China; 4 Laboratory Animal Center, Fujian Medical University, Fuzhou, Fujian, China; 5 School of Pharmacy, Fujian Medical University, Fuzhou, Fujian, China; 6 Laboratory of Snake Venom, The Center of Translational Hematology, Fujian Medical University, Fuzhou, Fujian, China; University of California Santa Barbara, UNITED STATES OF AMERICA

## Abstract

Over the years, CAR-T cell therapy has achieved remarkable success in treating hematological malignancies. However, this efficacy has not been replicated in the context of glioblastoma (GBM). In this study, a PD1CD28 chimeric molecule was introduced into EGFRvⅢ-directed CAR-T cells, generating EGFRvⅢ-P2A-PD1CD28 CAR-T cells. Notably, this modification significantly increased IL-2 secretion and enhanced antigen-dependent activation of CAR-T cells, especially when programmed cell death ligand 1 (PD-L1) was present *in vitro*. In addition, the *in vivo* xenograft experiments revealed that the PD1CD28 chimeric molecule played a pivotal role in reducing recurrence rates, effectively controlling recurrent tumor volume, and ultimately prolonging the survival of mice. Collectively, these findings suggest that EGFRvⅢ-directed CAR-T cells co-expressing the PD1CD28 chimeric molecule have the potential to significantly enhance the treatment efficacy against GBM.

## Introduction

Glioblastoma, a highly aggressive and prevalent malignant brain tumor mainly affecting adults, poses significant therapeutic challenges during clinical practice despite unprecedented scientific progress achieved in recent years [1]. Existing treatment options include radiotherapy, chemotherapy, surgical resection and tumor-treating field therapy [2]. Despite the application of aggressive multimodal treatments, approximately 70% of GBM patients experience disease progression within one year [3]. Moreover, the five-year survival rate for GBM patients remains dismally low, not exceeding 5% [4]. Given the poor prognosis of conventional treatments, the spotlight has shifted towards emerging therapies like chimeric antigen receptor T-cell therapy [5].

It is now understood that the conventional chimeric antigen receptor (CAR) structure in CAR-T cells comprises a single-chain variable fragment (scFv) responsible for recognizing tumor-associated antigens, one or more intracellular costimulatory domains, and a CD3ζ derived from the T cell receptor (TCR). Importantly, the CAR’s structure enables CAR-T cells to target and eliminate tumor cells independently of major histocompatibility complex (MHC) recognition achieved through the direct interaction between the scFv and tumor-associated antigens, thus overcoming immune evasion often linked to low MHC expression within tumor sites [6]. Past clinical trials have showcased significant success with CAR-T cell therapy in hematological malignancies. Furthermore, there is evidence supporting the efficacy of CAR-T cells targeting EGFRvⅢ in GBM treatment [7–9]. Despite these advancements, tumor recurrence remains an issue attributed to the immunosuppressive tumor microenvironment (TME) often observed in GBM patients [10–12]. A key player in this context is the upregulation of programmed cell death ligand 1 (PD-L1) expression in gliomas post-treatment, which subsequently triggers immune suppression and impairs CAR-T cell function through the PD-1/PD-L1 signaling axis [13, 14]. Given this scenario, targeting the PD-1/PD-L1 axis emerges as a logical approach for enhancing the therapeutic potential of CAR-T cells against GBM.

In the present study, EGFRvⅢ-targeted CAR-T cells were genetically modified to co-express a PD1CD28 chimeric molecule, which effectively replaces the PD-1 transmembrane and intracellular domains with a co-stimulatory CD28 domain. The objective was to create EGFRvⅢ-P2A-PD1CD28 CAR-T cells capable of effectively targeting and eliminating tumor cells expressing EGFRvⅢ. The introduced PD1CD28 chimeric molecules could bind to PD-L1 on tumor cells, which leads to a transformation of inhibitory signals into activating ones, thereby enhancing the overall function of T cells. This study sheds light on the characteristics of PD1CD28 chimeric molecules in conjunction with EGFRvⅢ-directed CAR-T cells, with the primary aim of restoring the functionality of EGFRvⅢ-targeted CAR-T cells by intervening in the PD-1/PD-L1 axis.

## Material and methods

### Mice and cell lines

Immune compromised NCG mice were purchased from Model Animal Resource Information Platform of Nanjing University and bred under pathogen-free conditions, according to NIH guidelines and were approved by the Institutional Animal Care and Use Committees of Fujian Medical University. The animal experiment researchers had received training and obtained the laboratory animal practitioner certificate. EGFRvⅢ^+^ U87 MG cell line overexpressing EGFRvⅢ artificially and WT U87 MG cell line were donated from Ludwig Institute, University of California. 293T cells were acquired from TaKara. Tumor cells and 293T cells were maintained in dulbecco’s modified eagle medium (Corning) supplemented with 10% heat inactivated fetal calf serum. T cells were maintained in RPMI-1640 (Corning) supplemented with 10% heat inactivated fetal calf serum, 1% L-glutamine, 30 IU/mL rhIL-2(Peprotech), 100 U/ml penicillin and100 μg/ml streptomycin sulfate. The PD-L1 KO EGFRvⅢ^+^ U87 MG cell line was generated by lentiviral transduction with CRISPR/Cas9 technology. Briefly, three sgRNAs targeting PD-L1 were designed, double-stranded sgRNA was formed and subcloned into the lentiCRISPRv2 vector. The primers were as follows: sgRNA-1 forward, 5′-CACCGTCCAGATGACTTCGGCCTT -3′, sgRNA-1 reverse, 5′-AAACAAGGCCGAAGTCATCTGGAC-3′, sgRNA-2 forward 5′-CACCGTGTCCAGATGACTTCGGCCT -3′, sgRNA-2 reverse, 5′-AAACAGGCCGAAGTCATCTGGACAC -3′, sgRNA-3 forward,5′-CACCGTCCAGATGACTTCGGCCTTG -3′and sgRNA-3 reverse, 5′-AAACCAAGGCCGAAGTCATCTGGAC -3′. Their gene editing efficiencies in EGFRvⅢ^+^ U87 MG cells were evaluated using flow cytometry by transient transfection of target cells with corresponding lentiCRISPRv2 plasmids. The highest gene editing efficiencies lentiCRISPRv2 vector was chosen and produced corresponding lentivirus particles. Subsequently, EGFRvⅢ^+^ U87 MG cell were transduced with these lentivirus particles. Two days after transfection, puromycin (2 μg/ml) was added to the culture medium to select target cells.

### Construction of the EGFRvⅢ CAR vectors

To generate EGFRvⅢ targeted CARs, EGFRvⅢ CAR was constructed by connecting the EGFRvⅢ specific scFv to a hinge and transmembrane domain of CD8, and the intracellular structure is composed of the intracellular domains of 4-1BB and CD3ζ in order. Anti-human EGFRvⅢ scFv was prepared according to previous reports [15, 16] with modifications. The extracellular domains of the PD1CD28 chimeric molecule correspond to those of PD-1, while the transmembrane and intracellular domain is substituted with a CD28 co-stimulatory domain [17]. The PD1CD28 chimeric molecule was subcloned into the lentiviral vectors (pLVX-EF1alpha-IRES-ZsGreen1, TaKara) downstream of a P2A sequence which was followed by the EGFRvⅢ CAR.

### Production of lentivirus

Lentivirus containing EGFRvⅢ CAR or EGFRvⅢ-P2A-PD1CD28 CAR were produced by transfection of 293T cells with plasmids expressing EGFRvⅢ CAR and the second generation packaging plasmids psPAX2 and pM D.2G on a 3:2:1 ratio using calcium phosphate cell transfection kit (Beyotime). The culture supernatants were collected and centrifuged to remove cell debris at 48 and 72 hours after transfection. The supernatants containing lentivirus were concentrated at 70,000 *g*, 4°C for 2 h with ultracentrifuge (Optima XPN-100, Beckman) after filtration through 0.45μm filters. Subsequently, the supernatant was discarded, 200 μL RPMI-1640 was added to the bottom of centrifuge tube and left at 4° C for 2 hours to facilitate virus particles resuspension. The concentrated lentivirus was titrated using 293 T cells and stored at −80°C. Briefly,Make a tenfold serial dilution of the retroviral preparation of the viral preparation in DMEM. Aim for a 2 μL volume of each dilution in each well of 24 well plate. After 48 hours, determine the percentage of CAR^+^ cells by flow cytometry. Calculate biological titer (BT = TU/ml) according to the following formula: TU/ml = (F × N × D × 1000/ V(μl), where F = % CAR^+^ cells (Choose 1~20%), N = number of cells at time of transduction, D = fold of dilution, V = volume of dilution added to each well.

### Isolation, activation and transduction of primary human T lymphocytes

Human peripheral blood mononuclear cells (PBMCs) from healthy donors were isolated using Ficoll-Paque PLUS (General Electric Company) following the manufacturer’s instructions, and then activated with anti-CD3/CD28 beads (Thermo Fisher Scientific) at a ratio of 1:1 (PBMCs: beads) following the manufacturer’s instructions (day 0). 48 h post-activation (day 2), the living cells were counted and transduced with lentivirus which pre-coated on the 6-well culture plates (MOI = 10) with 20μg/mL RetroNectin (Takara). To increase infection efficiency, the culture plate was centrifuged for 45 min at 800 *g*, 32°C after adding the cells. After co-incubation for 16 hours in incubator, cells were washed once and maintain in 1640-RIPM medium containing 30IU/mL rhIL-2. IL-2 was supplemented every two days to maintain the effective working concentration. Three days post-transduction, the expression rate of CAR and PD1CD28 chimeric molecule were analyzed by flow cytometry. Three days after transduction (day 5), CAR-T cells were cultured in the medium without IL-2 and anti-CD3/CD28 beads for one day to being used for functional assays in vitro. The CAR-T cells were cryopreserved at day 10 for animal experiments.

### CAR-T cells functional assays

The lytic ability of T cells (day 7) was analyzed by co-culturing corresponding T cells with CFSE (Invitrogen)-labeled target cells (EGFRvⅢ^+^ U87 MG, WT U87 MG or PD-L1 KO EGFRvⅢ^+^ U87 MG) on a 96-cell culture plate with round bottom at the indicated effector: target (E: T) ratios for 10 h. The cells were transferred to round-bottom tube, and 0.1 μg DAPI (Invitrogen) was added to the tube and vortex immediately, then analyzed by flow cytometry within 5s. The % CAR-T cells lysis were calculated as follows: (experimental CFSE^+^DAPI^+^ cells/ total CFSE^+^ cells −control CFSE^+^DAPI^+^ cells/total CFSE^+^ cells) ×100%, where “experimental” indicate the target cells with effector CTL while “control” represents the target cell alone without effector cells. Cytokine secretion of T cells (day 7) were determined by co-culturing 5×10^4^ CAR-T cells with 5×10^4^ target cells for 24 hours after removal of anti-CD3/CD28 beads for one day in advance. The supernatants were collected after centrifuging at 12,000 *g*, 4°C for 10 min. Cytokine levels of IFN-γ and IL-2 in the co-cultured supernatants were detected using the Human IFN-gamma Quantikine ELISA Kit (R&D Systems) and the Human IL-2 ELISA Kit Ⅱ (BD Biosciences) according to the manufacturer’s instructions. Intracellular staining assay for IL-2 was performed using Fixation/Permeabilization kit plus with BD Golgplug (BD Biosciences) to improve aggregation of cytokines, followed by labeling of IL-2 with PE mouse anti-human IL-2 (BD Biosciences). Briefly, add 1μL of BD Golgi Plug for every 1 mL of CAR-T cells (day 7) culture and mix thoroughly. Subsequently, 5×10^4^ CAR-T cells with 5×10^4^ target cells were co-cultured for 10 hours. The cells were incubated with anti-human IgG, F(ab’)₂ fragment specific antibodies for 30 min at 4°C, then thoroughly resuspend cells and add 100 μL per well for microwell plates of Fixation/ Permeabilization solution for 20 minutes at 4°C. Finally, resuspend fixed/permeabilized cells in 50 μL of BD Perm/Wash™ buffer containing PE mouse anti-human IL-2 antibody, incubate at 4°C for 30 minutes in the dark. The proportion of CAR-T cells secreting IL-2 were analyzed by gating CAR^+^ T cells. For the measurement of CAR-T cell proliferation, CAR-T cells and mitomycin-C-treated target cells (5 μg/μL) were co-cultured on a 24-cell culture plate. The absolute number of T cells was determined using precision count beads (Biolegend) at 72 hours.

### Animal models and treatment

NCG mice were injected with 5×10^5^ EGFRvⅢ^+^ U87 MG cells in the flank subcutaneously, and the mice were randomized to each treatment groups (n = 6). When the tumors reached a volume of 50-100mm^3^ (V=π6×a×b2, a: length, b: width) at days 5–6 after injection. NCG mice bearing EGFRvⅢ^+^ U87 MG cells were treated with: CAR-T cells (EGFRvⅢ CAR-T, EGFRvⅢ-PD1CD28 CAR-T, 4×10^6^ CAR^+^ T cells per mouse by intravenous injection) and non-transduced T cells (an equivalent number per mouse (matched for total T cell dose) by intravenous injection respectively. Animal health and behavior were monitored every two days and the tumor size was measured every 2 days with calipers until the tumors reached a volume of 2000 mm^3^. 3 Mice in each group were sacrificed on Day 15 post-treatment and the tumors tissues were preserved for immunohistochemistry to quantify persistent and distribution of T cells.

Mice were sacrificed by intraperitoneal injection of 3% sodium pentobarbital at a dose of 50mg/kg when the predetermined humane endpoints were reached (tumors reached volume of 2000 mm^3^, the tumor site began to ulcerate or the mice rapidly lost 15~20% of their original body weigh) or the endpoint of the experiment was reached. Once animals reached endpoint criteria, they were euthanized immediately. None of the animals died before meeting criteria for euthanasia. The duration of the experiment was 120 days.

### Flow cytometry and immunohistochemistry

The expression levels of EGFRvⅢ were determined by anti- EGFRvⅢ monoclonal antibody (abcam). The expression of CAR molecule and PD1CD28 chimeric molecule were analyzed using AffiniPure F(ab’)₂ Fragment Goat Anti-Human IgG, F(ab’)₂ fragment specific(Jackson ImmunoResearch) and anti-human PD-1 antibody (BD Biosciences). The proportion of activation of T cells was detected with FITC-labeled anti-human CD25 antibody and APC-labeled anti-human CD69 antibody (BD Biosciences). Each reaction was incubated with specific fluorescent-labeled (100 μL) at 4°C for 30 min, washed three times and run on FACS Calibur or FACS Verse (BD Biosciences). Mouse tumor tissues were analyzed for CD3 expression (anti-human CD3, Abcam). All IHC staining performed according to the protocol described previously [18]. ImageJ version 1.8.0 software was used to assess the area and density of the dyed region, and the integrated optical density (IOD) value of the IHC section. The average optical density (AOD) of the digital image (magnification, ×200) was designated as representative CD3 staining intensity (indicating the relative CD3 expression level).

### Statistical analysis

Statistical evaluation was analysis by using GraphPad Prism software v.6.0. Data are shown of a minimum of three biological replicates or independent experiments. All data are presented as the means ± SD. P values < 0.05 were considered to be significant.

## Results

### Anti-EGFRvⅢ CAR-T cells co-expressing PD1CD28 chimeric molecule demonstrate augmented IL-2 secretion

**Fig 1:**
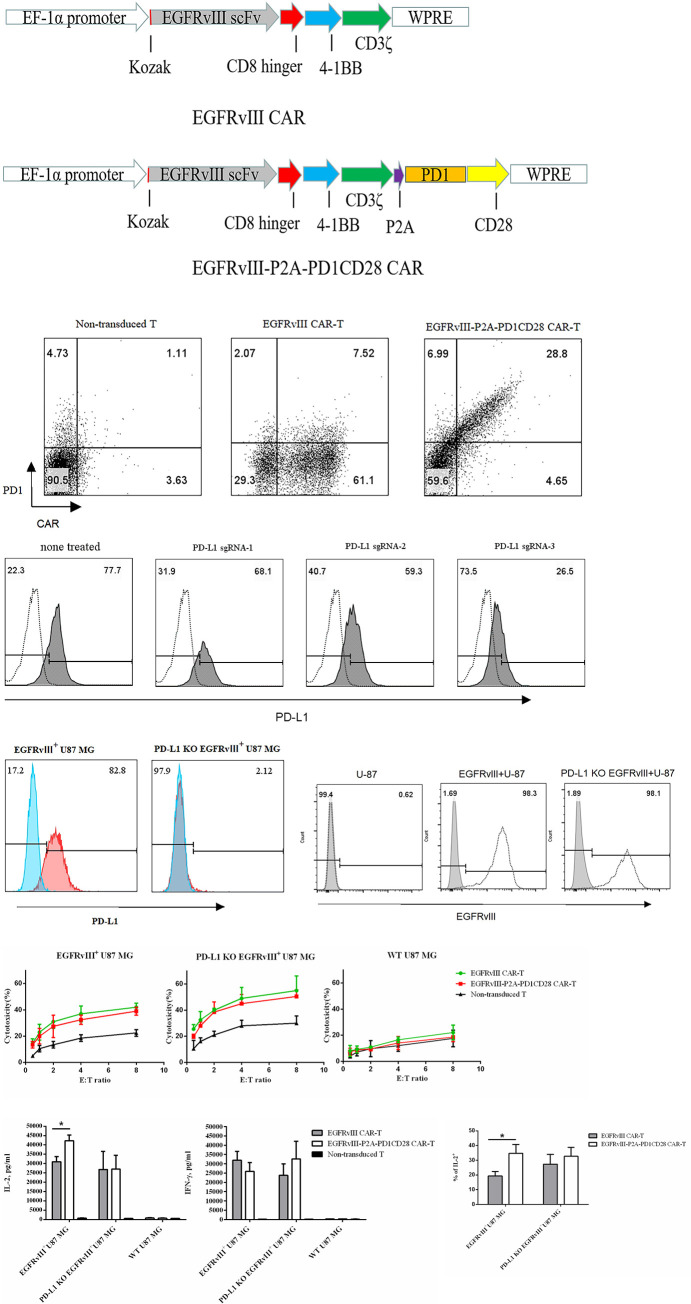
EGFRvⅢ-P2A-PD1CD28 CAR-T cells produce higher level of IL-2. (A) The schematic diagram of lentivirus construction of EGFRvⅢ CAR co-expressing PD1CD28 chimeric molecule. (B) Flow cytometry analysis of CAR and PD1CD28 chimeric molecule expressed on T cells which transduced with lentivirus. The expression of CAR was detected using the anti-human IgG F(ab’)₂ antibody, and PD1CD28 chimeric molecule was determined with anti-human PD1 antibody. (C) The knockout efficiencies of different sgRNA were determined by detecting the PD-L1expression level of EGFRvⅢ^+^ U87 MG cells. (D) The expression level of PD-L1 of EGFRvⅢ^+^ U87 MG cells and PD-L1 KO EGFRvⅢ^+^ U87 MG cells was determined by flow cytometry using anti-human PD-L1 antibody. (E) The expression level of EGFRvⅢ of WT U87 MG cells, EGFRvⅢ^+^ U87 MG cells and PD-L1 KO EGFRvⅢ^+^ U87 MG cells was determined by flow cytometry using anti-human EGFRvⅢ monoclonal antibody. (F) EGFRvⅢ CAR-T cells, EGFRvⅢ-P2A-PD1CD28 CAR-T cells or non-transduced T cells were co-cultured with EGFRvⅢ^+^ U87 MG cells (left), PD-L1 KO EGFRvⅢ^+^ U87 MG cells (middle), WT U87 MG cells (right) for 10 hours respectively. The lysis of tumor cells by CAR-T cells and non-transduced T cells were analyzed. (G) ELISA was performed to measure the levels of IL-2 (left) and IFN-γ (right) present in the supernatants after two types of CAR-T cells were cocultured with EGFRvⅢ^+^ U87 MG cells or PD-L1 KO EGFRvⅢ^+^ U87 MG cells at a 1:1 E:T ratios for 24 hours. (H) IL-2 secretion of CAR-T cells was measured by intracellular staining with the same co-culture method mentioned above. The percentage of cells expressing IL-2 in the CAR^+^ gates was analyzed. Data are the means ±SD of 3 independent experiments. *p<0.05.

The schematic diagram of lentivirus construction of EGFRvⅢCAR co-expressing PD1CD28 chimeric molecule was shown in Fig 1A. Human T cells were successfully transduced using a lentivirus with a second-generation construct that co-expressed anti-EGFRvⅢ CAR and the PD1CD28 chimeric molecule at a 1:1 ratio (Fig 1B). Initially, EGFRvⅢ^+^ U87 MG cells naturally expressed PD-L1, prompting us to create a PD-L1 knockout cell line termed PD-L1 KO EGFRv^+^ U87 MG cells, utilizing the CRISPR/Cas9 technology. The knockout efficiency of sgRNA-3 was greater than that of others in EGFRvⅢ^+^ U87 MG cells (Fig 1C). Thus, we decided to continue our studies using sgRNA-3. Next, we delivered CRISPR/Cas9 gene editing components guided by sgRNA-3 into EGFRvⅢ^+^ U87 MG cells using a lentiviral vector system. Two days after transduction, puromycin was added to the culture medium for one week to select PD-L1 KO EGFRvIII^+^ U87 (Fig 1D). We detected the EGFRvⅢ expression levels among wild-type U87, EGFRvIII^+^ U87 and PD-L1 KO EGFRvIII^+^ U87 (Fig 1E). In order to detect the cytotoxicity of anti-EGFRvⅢ CAR-T cells, anti-EGFRvⅢ CAR-T cells with and without the PD1CD28 chimeric molecule were co-cultured with different types of tumor cells, including WT U87 MG cells, EGFRvⅢ^+^ U87 MG cells and PD-L1 KO EGFRvⅢ^+^ U87 MG cells, at E:T ratios of 0.5:1 to 8:1(Fig 1F). As expected, when co-cultured with WT U87MG cells, both types of CAR-T cells showed no significant cytokine release or specific killing activity (Fig 1F, right graph; Fig 1G). However, when exposed to EGFRvⅢ^+^ U87 MG cells expressing both EGFRvⅢ and PD-L1, both types of CAR-T cells exhibited similar levels of lytic activity and IFN-γ release (Fig 1F, left graph; Fig 1G, right graph). Interestingly, CAR-T cells co-expressing the PD1CD28 chimeric molecule (EGFRvⅢ-P2A-PD1CD28 CAR-T cells) displayed a greater secretion of IL-2 (Fig 1G, left graph; Fig 1H). Conversely, when co-cultured with PD-L1 KO EGFRvⅢ^+^ U87 MG cells, there was no significant difference in IL-2 secretion between the two CAR-T cell types.

### Anti-EGFRvⅢ CAR-T cells co-expressing PD1CD28 chimeric molecule demonstrate augmented antigen-dependent activation and T cells proliferation

**Fig 2:**
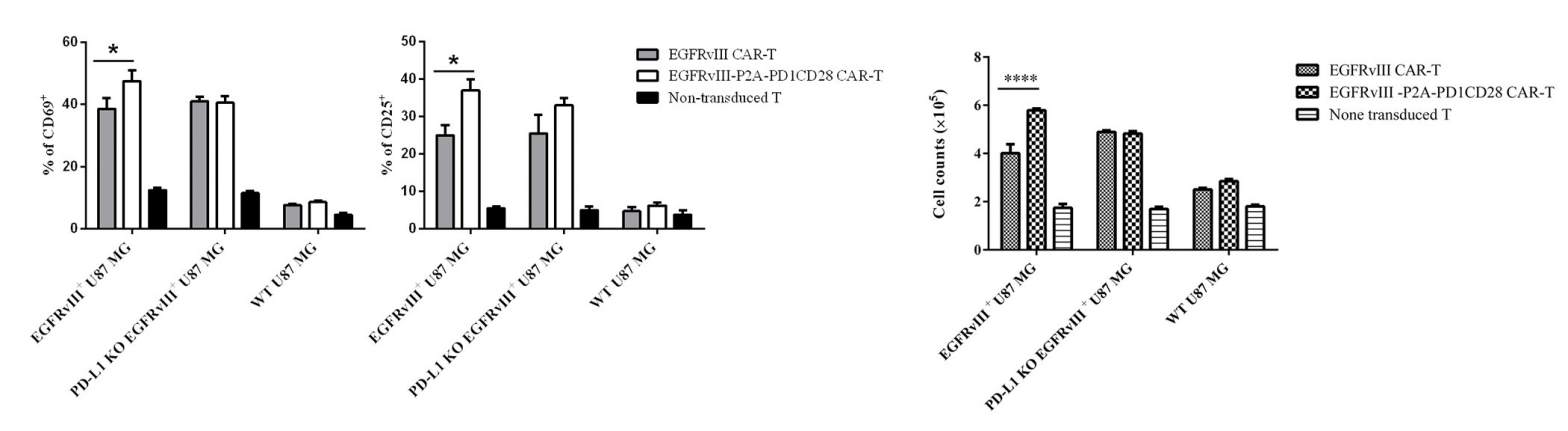
EGFRvⅢ-P2A-PD1CD28 CAR-T cells showed stronger capacity of antigen-dependent activation and proliferation. (A) Proportion of CD69^+^ T cells(left) or CD25^+^ T cells(right) after co-culture with different tumor cells for 24 h. (B) For the measurement of CAR-T cell proliferation, CAR-T cells and mitomycin-C-treated target cells were co-cultured. The absolute number of T cells was determined using precision count beads at 72 hours. Data are the means ±SD of 3 independent experiments. *p<0.05, ****p<0.0001.

**Fig 3:**
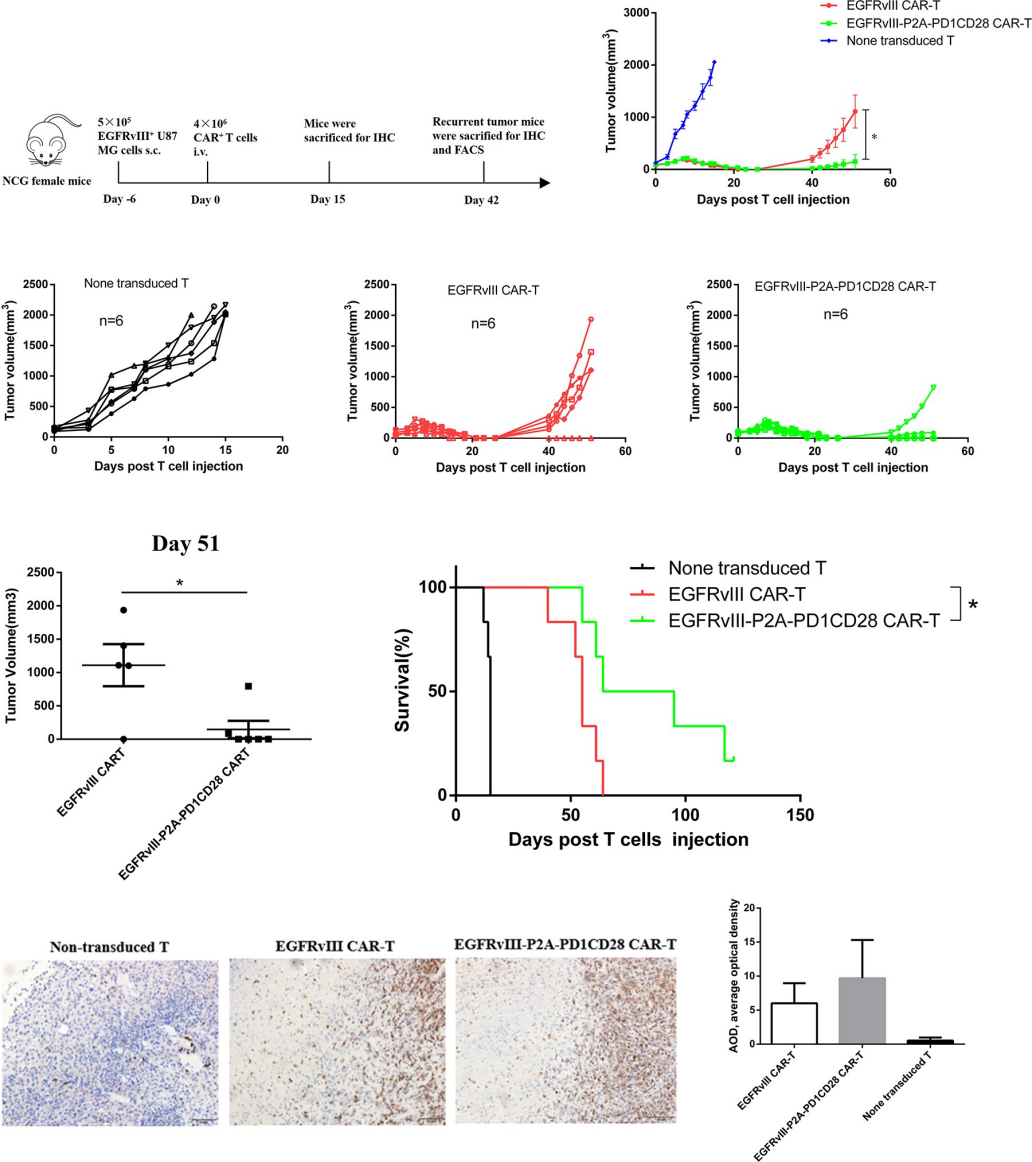
PD1CD28 chimeric molecule were tested in glioblastoma tumor engrafted NCG mice. (A) Schematic representation of in vivo antitumor experiment. (B-D) NCG mice (n = 6) were implanted in the flanks with EGFRvⅢ^+^ U87-MG tumor cells (5×10^5^ cells/mouse, s.c.) and were treated with a single dose of administration of anti-PD1 antibody alone, EGFRvⅢ CART cells i.v. ± i.p. administration of anti-PD1 antibody (10 mg/kg, 5 days after injection of CART cells), EGFRvⅢ-P2A-PD1CD28 CART cells at day 6 after tumor inoculation. Injection of non-transduced T cells served as control. Tumor sizes were measured, and the volumes of tumor were calculated and plotted by day 51. (E) The percentage survival per group was determined on a daily basis and is represented in a Kaplan–Meier survival curve. (F) Representative IHC images were shown at 200×magnification to determine the expression of CD3. Day 15 post-treatment, mice from each treatment group were sacrificed, and immunohistochemistry analysis was followed. Human T cells were detected by anti-human CD3 antibody and were stained in brown. (G) The average optical density (AOD) of the immunohistochemistry image (magnification, ×200) was designated as representative CD3 staining intensity, indicating the relative numbers of CD3^+^ TILs.

As expected, in the presence of EGFRvⅢ^+^ U87 MG cells, the proportion of CD25^+^ T cells and CD69^+^ T cells in the EGFRvⅢ-P2A-PD1CD28 CAR-T cell group was significantly higher than in the conventional CAR-T cell group (Fig 2A). This difference, however, was not observed when co-cultured with PD-L1 KO EGFRvⅢ^+^ U87 MG cells. To elucidate the effect of PD1CD28 chimeric molecule on the proliferation of CAR-T cells, we co-cultured EGFRvⅢ CAR-T cells and EGFRvⅢ-P2A-PD1CD28 CAR-T cells with mitomycin C-treated EGFRvⅢ^+^ U87 MG cells for 3 days. Notably, the number of CAR^+^ T cells in the 15 × 19 EGFRvⅢ CAR group exhibited a significantly higher compared to the EGFRvⅢ CAR group on day 3 (Fig 3B). Consistently with other results, when co-cultured with PD-L1 KO EGFRvⅢ^+^ U87 MG cells, there was no significant difference in T cells proliferation between the two CAR-T cell types.

### PD1CD28 chimeric molecule enhances in-vivo tumor control of EGFRvⅢ^+^ U87 MG cells flank tumors

To assess the impact of the PD1CD28 chimeric molecule, experiments were conducted using EGFRvⅢ^+^ U87 MG cells in a flank tumor-bearing NCG mouse model (Fig 3A). When the tumor reached a certain volume (50–100 mm^3^), tumor-bearing NCG mice were treated with non-transduced T cells, EGFRvⅢ CAR-T cells and EGFRvⅢ-P2A-PD1CD28 CAR-T cells i.v., all treatment for one dose. Mice injected with non-transduced T cells exhibited similar rates of tumor volume growth. In contrast, CAR-T cell treatment led to a notable deceleration in tumor growth by day 15 (Fig 3B and 3C). However, there was no significant difference in the reduction of tumor volume among the groups receiving EGFRvⅢ CAR-T cells and EGFRvⅢ-P2A-PD1CD28 CAR-T cells. Furthermore, by day 21, tumors in all CAR-T cell groups demonstrated regression (tumor can’t be subcutaneously touched). However, by day 28 post-treatment, 3 of 6 (50%) of the EGFRvIII CAR-treated mice and 0 of 6 (0%) of the EGFRvIII-P2A-PD1CD28 CAR-T-treated mice displayed tumor recurrence. On day 51 post-treatment, the recurrence rate for the EGFRvIII-P2A-PD1CD28 CAR-T cell group was 33%, while the EGFRvIII CAR-T cell group exhibited a recurrence rate of 67%. Notably, the tumor volume of the EGFRvIII-P2A-PD1CD28 CAR-T cell group was significantly smaller than that of the EGFRvIII CAR-T cell group on day 51 (Fig 3D). Furthermore, the median survival time of the EGFRvIII-P2A-PD1CD28 CAR-T cell group (79.5 days) exceeded that of the EGFRvIII CAR-T cell group (55 days) (Fig 3E), which suggests that the EGFRvIII-P2A-PD1CD28 CAR-T cell group effectively reduced recurrence rates, controlled recurrent tumor volume, and prolonged the survival of EGFRvⅢ^+^ U87 MG cells in the flank tumor-bearing NCG mice. Immunohistochemistry results showed that CAR-T cells injected into the tail vein could reach the tumor site (Fig 3F). In addition, although the numbers of CD3^+^ TILs did not significantly differ between the two CAR-T cell types, EGFRvIII-P2A-PD1CD28 CAR-T cells displayed a trend toward greater numbers compared to EGFRvⅢ CAR-T cells (Fig 3G).

### Analyses of tumor recurrent of EGFRvⅢ^+^ U87 MG cells flank tumors human glioblastoma

**Fig 4:**
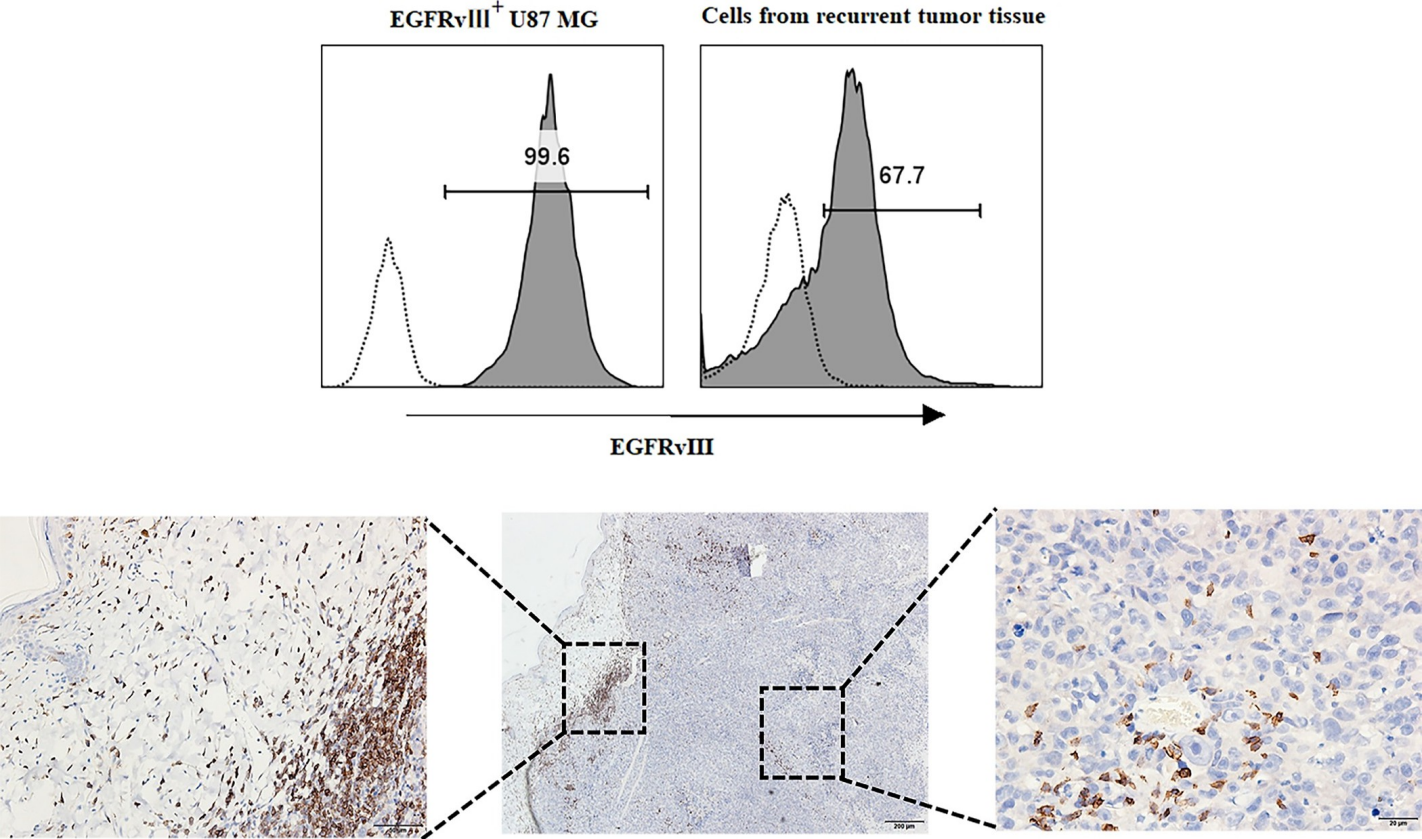
Analyses of tumor recurrent of EGFRvⅢ^+^ U87 MG cells flank tumors. Day 42 post-treatment, the recurrent tumor mice were sacrifice, flow cytometry (A) and immunohistochemistry (B) were done. Anti-human EGFRvⅢ antibody was used to detect whether EGFRvⅢ is still expressed on recurrent tumor cells. Anti-human CD3 antibody was used to determine the distribution of human T cells in recurrent tumor tissues.

Regarding the cause of tumor recurrence, we suspect that the loss of tumor-specific antigens is responsible. Flow cytometry analysis of recurrent tumor cells indicated the persistence of EGFRvⅢ expression, but at a decreased abundance (Fig 4A). Immunohistochemistry results of recurrent tumor revealed that T cells were predominantly located at the periphery of recurrent tumors, with few T cells observed in the core region (Fig 4B)

## Discussion

In the current study, the PD1CD28 chimeric molecule was introduced to EGFRvⅢ-targeting CAR-T cells to generate a novel CAR-T cells for the first time. Compared to conventional EGFRvⅢ-specific CAR-T cells, EGFRvIII-P2A-PD1CD28 CAR-T cells exhibited enhanced functionality and anti-tumor effects against PD-L1^+^/EGFRvⅢ^+^ tumors.

Around 30% of newly diagnosed GBM patients were found to express EGFRvⅢ [19], which enhanced glioblastoma cell proliferation and hindered apoptosis [20–22]. Notably, EGFRvⅢ is exclusively expressed in tumor cells [23], indicating its role as a tumor-specific antigen and an ideal target for CAR-T cell therapy in GBM. Safety assessments have shown no evidence of cross-reactivity to wild-type EGFR or neurological toxicity in clinical trials, although local cytokine release was noted after EGFRvⅢ-targeting CAR-T cell infusions [10, 14]. Nonetheless, some clinical trials have indicated limited efficacy of EGFRvⅢ-CAR-T cells in treating GBM [11, 14, 24]. Factors such as specific loss or reduced expression of EGFRvⅢ, tumor heterogeneity, and the immunosuppressive TME may significantly restrict the potential of CAR-T cell therapy for GBM.

Several studies have unveiled that the function of CAR-T cells could be compromised by the PD-1/PD-L1 axis activated in the tumor microenvironment, constituting an established immune escape mechanism [25–29]. Overcoming the immunosuppressive TME could potentially enhance the therapeutic effectiveness of CAR-T cell therapy for solid tumors. In recent years, diverse strategies have been designed to target the PD-1/PD-L1 axis in CAR-T cell therapy, including CRISPR-Cas9-mediated PD-1 knockout in CAR-T cells, combining CAR-T cells with anti-PD1 antibodies and generating human soluble PD-1 produced by CAR-T cells [13, 30, 31]. Additionally, the PD1CD28 chimeric molecule has been successfully applied to enhance the performance of anti-PSCA, anti-B7H3, and anti-CD22 CAR-T cells [17, 32, 33]. Notably, this study represents the first exploration of co-expressing the PD1CD28 chimeric molecule in anti-EGFRvⅢ CAR-T cells, specifically tested within a GBM model.

Our study provided compelling evidence of a significant difference in IL-2 secretion only when EGFRvⅢ-directed CAR-T cells were co-cultured with tumor cells expressing PD-L1. We hypothesized that the extracellular domain of the PD1CD28 chimeric molecule’s PD-1 could bind to PD-L1 expressed on tumor cells, triggering activation of the intracellular co-stimulatory signaling domain of CD28 in the PD1CD28 chimeric molecule [17]. This subsequently led to a notable increase in IL-2 secretion. Consistently, when co-cultured with PD-L1-expressing tumor cells, the EGFRvⅢ-P2A-PD1CD28 CAR-T cell group demonstrated a higher proportion of activated T cells, indicating a stronger ability for antigen-dependent CAR activation. In addition, EGFRvⅢ-P2A-PD1CD28 CAR-T cells showed stronger capacity of proliferation with antigenic stimulation.

Like other malignant tumors that express PD-L1, GBM often exhibits frequent PD-L1 expression levels within certain subpopulations [34]. Some studies have even reported PD-L1 expression in over 88% of GBM patients [35]. Consistently, EGFRvⅢ^+^ U87 MG cells used in our study naturally express high PD-L1. However, in most of the previous studies about PD1CD28 chimeric molecule [17, 33, 36], PD-L1 was lentivirally transduced into the tumor cell lines to produce high stable expression of PD-L1 version, due to the tumor cell lines expressed low levels of PD-L1 naturally. Moreover, we further validated the results from previous studies by knocking out PD-L1 in tumor cells, PD1CD28 chimeric molecule expression enhanced IL-2 secretion and T cells proliferation in a PD-L1-dependent manner. Therefore, we posit that the application of the PD1CD28 chimeric molecule to EGFRvⅢ-directed CAR-T cells could hold more significance than those aimed at tumor cells expressing little to no PD-L1. The major risks of CAR-T cell therapy are the cytokine syndrome and on-target, off-tumor toxicity [37, 38]. Consistent with previous studies, we found that PD1CD28 chimeric molecule augments the cytotoxicity efficacy of CAR-T cells *in vivo*. Given that, when on-target, off-tumor effect occur, CAR-T cells co-expressing PD1CD28 chimeric molecules will cause greater damage to normal cells and face higher levels of cytokine release compare to conventional CAR-T cells, in theory. Notably, differ from other studies, EGFRvⅢ is a tumor-specific antigen so that the probability of on-target, off-tumor toxicity is extremely low.

Regrettably, despite their ability to reach the tumor site, control tumor volume, and prolong the survival of EGFRvⅢ^+^ U87 MG cells in the flank tumor-bearing NCG mice, CAR-T cells co-expressing PD1CD28 still showed tumor recurrence, which could be attributed to decreased antigen expression and the CAR-T cells’ inability to effectively infiltrate the tumor’s core. Further investigations are warranted to comprehensively understand the causes of recurrence, such as the phenotypes of dysfunctional tumor-infiltrating T lymphocytes (TILs), and other factors driving their dysfunction. Similarly, the immunophenotypes of CAR-T cells *in vitro* also need to be further explored to illuminate the *in vivo* findings. In addition, we acknowledge the lack of an appropriate control group of T cells expressing PD1CD28 chimeric molecules alone in our study design, which hindered our ability to validate the effect of the PD1CD28 chimeric molecule on T cell immunophenotypes. Moreover, it should be borne in mind that this study evaluated the impact of the PD1CD28 chimeric molecule using a flank tumor-bearing mice model. This model, however, may not accurately replicate the process of T cells entering the tumor site through the blood-brain barrier, as seen in an *in situ* model [14, 39]. Given the unique TME in the brain, which differs from that of subcutaneous tumors, establishing an intracranial orthotopic model presents challenges. Thus, having proven its efficacy in the flank tumor-bearing model, we intend to conduct *in situ* experiments to further explore the role of PD1CD28 chimeric molecule.

### Conclusions

In summary, our findings offer compelling evidence that the co-expression of the PD1CD28 chimeric molecule in EGFRvⅢ-directed CAR-T cells can outperform conventional CAR-T cells in PD-L1^+^/EGFRvⅢ^+^ tumors both *in vitro* and *in vivo*. Consequently, we advocate for the introduction of the PD1CD28 chimeric molecule to EGFRvⅢ-targeted CAR-T cells in future clinical trials for GBM treatment.

## Supporting information

S1 Data Minimal data set containing the underlying numerical data to generate Figs 1–4. (ZIP)
